# Alogliptin prevents diastolic dysfunction and preserves left ventricular mitochondrial function in diabetic rabbits

**DOI:** 10.1186/s12933-018-0803-z

**Published:** 2018-12-27

**Authors:** Xiaowei Zhang, Zhiwei Zhang, Yajuan Yang, Ya Suo, Ruimeng Liu, Jiuchun Qiu, Yungang Zhao, Ning Jiang, Changle Liu, Gary Tse, Guangping Li, Tong Liu

**Affiliations:** 1 Tianjin Key Laboratory of Ionic-Molecular Function of Cardiovascular Disease, Department of Cardiology, Tianjin Institute of Cardiology, Second Hospital of Tianjin Medical University, No. 23 Pingjiang Road, Hexi District, Tianjin, 300211 People’s Republic of China; 2 Tianjin Key Laboratory of Exercise Physiology and Sports Medicine, Department of Health & Exercise Science, Tianjin University of Sport, Tianjin, 300381 People’s Republic of China; 3 Department of Medicine and Therapeutics, Chinese University of Hong Kong, Hong Kong, SAR, China; 4 Li Ka Shing Institute of Health Sciences, Chinese University of Hong Kong, Hong Kong, SAR, China

**Keywords:** Dipeptidyl peptidase-4 inhibitors, Diabetic cardiomyopathy, Diabetes mellitus, Mitochondrial function, Mitochondrial biogenesis

## Abstract

**Background:**

There are increasing evidence that left ventricle diastolic dysfunction is the initial functional alteration in the diabetic myocardium. In this study, we hypothesized that alogliptin prevents diastolic dysfunction and preserves left ventricular mitochondrial function and structure in diabetic rabbits.

**Methods:**

A total of 30 rabbits were randomized into control group (CON, n = 10), alloxan-induced diabetic group (DM, n = 10) and alogliptin-treated (12.5 mg/kd/day for 12 weeks) diabetic group (DM-A, n = 10). Echocardiographic and hemodynamic studies were performed in vivo. Mitochondrial morphology, respiratory function, membrane potential and reactive oxygen species (ROS) generation rate of left ventricular tissue were assessed. The serum concentrations of glucagon-like peptide-1, insulin, inflammatory and oxidative stress markers were measured. Protein expression of TGF-β1, NF-κB p65 and mitochondrial biogenesis related proteins were determined by Western blotting.

**Results:**

DM rabbits exhibited left ventricular hypertrophy, left atrial dilation, increased E/e′ ratio and normal left ventricular ejection fraction. Elevated left ventricular end diastolic pressure combined with decreased maximal decreasing rate of left intraventricular pressure (− dp/dtmax) were observed. Alogliptin alleviated ventricular hypertrophy, interstitial fibrosis and diastolic dysfunction in diabetic rabbits. These changes were associated with decreased mitochondrial ROS production rate, prevented mitochondrial membrane depolarization and improved mitochondrial swelling. It also improved mitochondrial biogenesis by PGC-1α/NRF1/Tfam signaling pathway.

**Conclusions:**

The DPP-4 inhibitor alogliptin prevents cardiac diastolic dysfunction by inhibiting ventricular remodeling, explicable by improved mitochondrial function and increased mitochondrial biogenesis.

## Introduction

Diabetes mellitus (DM) is a global epidemic with significant adverse impacts on life expectancy and quality of life. More than 170 million people worldwide suffer from diabetes, and this figure is projected to double by the year 2030 [1]. It is widely recognized that DM is associated with adverse alterations in myocardial structure and function, collectively known as diabetic cardiomyopathy, which is responsible for the development of heart failure in this population [2–4]. One of the hallmarks of diabetic cardiomyopathy is left ventricular diastolic dysfunction, which is often associated with normal systolic function in the early disease processes [5]. Indeed, the incidence of heart failure with preserved ejection fraction (HFpEF) in diabetes has increased sharply over the past two decades [6–8].

Diastolic dysfunction is characterized by increased myocardial stiffness leading to abnormal left ventricular relaxation that is associated with increased filling pressure. Poor glycemic control adversely affects diastolic function by several mechanisms, including increased oxidative stress, chronic inflammation, myocardial hypertrophy, perivascular and interstitial collagen deposition and cross-linking, abnormal intracellular calcium handling, endothelial and mitochondrial dysfunction and apoptosis [9–12]. The mitochondria are the main sites where reactive oxygen species (ROS) are generated [13]. Previously, our group found that the atrial tissue of diabetic rabbits showed markedly higher mitochondrial ROS generation rate in contrast with the control group, and the treatment of dipeptidyl peptidase-4 (DPP-4) inhibitor, alogliptin, suppressed their generation and attenuate adverse atrial remodeling [14]. By contrast, the effects of DPP-4 inhibitors on ventricular function in diabetes are not completely understood. Therefore, in this study, we aimed to investigated the mechanisms by which alogliptin affects ventricular function in early stage of diabetes specifically focusing on the role of the mitochondria.

## Methods

### Experimental animals and study protocol

This study was approved by the Experimental Animal Administration Committee of Tianjin Medical University and Tianjin Municipal Commission for Experimental Animal Control. Japanese white rabbits (1.8–2.2 kg) were purchased from Beijing Medical Animals Research Institute. A computer was used to generate 30 different random numbers corresponding to every rabbit. The first 10 rabbits were taken as the control group, the middle 10 rabbits as the DM group, and the remaining 10 rabbits as the alogliptin-treated DM (DM-A) group. In each group, echocardiographic, hemodynamic, histological, serum biochemical and oxidative stress–related markers, as well as Western blot and mitochondrial function were determined.

### Rabbit model of diabetes mellitus

The alloxan-induced diabetes model using rabbits has been previously used by our group [15, 16]. Briefly, it involves intravenous injection of 5% alloxan (alloxan monohydrate, Sigma Aldrich Chemical; 120 mg/kg) dissolved in sterile normal saline into the marginal ear vein. This produces a type 1 diabetes mellitus model by damaging the pancreatic beta cells. Fasting blood glucose was measured 48 h later with confirmation of diabetes by glucose levels ≥ 14 mmol/L. If the fasting blood glucose level did not reach diagnostic criteria after 48 h, then the same dose of alloxan was injected again. If this failed to induce diabetes again, then the rabbit was excluded from the study. Fasting blood glucose concentration was monitored weekly using the glucometer Optium Xceed (Abbott Labora-tories MediSense Products). After diabetes was established, animals in the DM-A group were given alogliptin (12.5 mg/kg/day; Tianjin Takeda Pharmaceuticals Co, Ltd) that was mixed into the food for 12 weeks. This dose was selected as it is in keeping with previously used dosing regimen in prior experiments [17].

### Echocardiographic assessment

After 12 weeks, all the rabbits underwent resting transthoracic two-dimensional echocardiography and Doppler imaging to assess left ventricular function by operators blinded to grouping. They were anesthetized with 3% pelltobarbitalum natricum (30 mg/kg), and echocardiographic parameters were obtained in the parasternal long-axis view using a GE Vingmed machine (Vivid 7/Vingmed General Electric) equipped with a 7.5-MHz standard pediatric probe. Standard imaging planes, M-mode, color-mode, Doppler, and functional calculations were acquired according to the guidelines of the American Society of Echocardiography [18]. Left atrial (LA) anteroposterior diameter, left ventricular posterior wall (LVPW) thickness, interventricular septal thickness (IVS), left ventricular end-diastolic dimension (LVEDD), and left ventricular end-systolic dimension (LVESD) were measured using both 2-dimensional and M-mode imaging during 5 consecutive cardiac cycles. Left ventricular ejection fraction was calculated, and the mean of three measurements was calculated for subsequent analysis. Left ventricular diastolic function was evaluated by the mitral peak velocity of early filling (E) to early diastolic mitral annular velocity (e′) (E/e′) ratio.

### Hemodynamic study and sample collection

After echocardiographic examination, each rabbit underwent right carotid artery cannulation for measuring hemodynamic parameters during ECG monitoring. Heart rate (HR), aortic systolic blood pressure (SBP), diastolic blood pressure (DBP), and mean blood pressure (MBP) were recorded carefully after a stabilization period using a BL-420F biological function detection system (Chengdu Taimeng Science and Technology Co, Ltd). Then the cannula was inserted through the aortic valve to the left ventricle to measure the ventricular end-diastolic pressure (LVEDP), maximal and minimal rates (± dp/dtmax) of the rise in left ventricular pressure.

Blood samples were obtained from the carotid artery after hemodynamic examination for measurements of serum biochemical, inflammatory, and oxidative stress markers. The animals were then euthanized and a portion ≈ 100 mg LV tissues were collected for mitochondrial function study immediately. Another about 100 mg LV tissues were frozen in liquid nitrogen, and stored at − 80 °C for western blot. Small pieces of LA tissue were immersed in 10% formaldehyde and 2.5% glutaraldehyde for histological and ultrastructural studies respectively.

### Isolation of mitochondria from the left ventricle

LV tissue collected as mentioned above was quickly dissected and minced in an ice-cold isolation medium containing mannitol 220 mmol/L, sucrose 70 mmol/L, HEPES 5 mmol/L, PMSF 1 mmol/L, BSA 0.2% (w/v), and pH 7.4. The minced blood-free tissue was homogenized using a manual glass homogenizer with 6 passes (0–4 °C). The homogenate was centrifuged at 1000×*g* for 10 min and the liquid supernatant was collected. It was then centrifuged at 10,000×*g* for 10 min. The major constituent of the deposit was mitochondrial pellet, which was suspended in 0.5 mL of the conversational medium containing mannitol 220 mmol/L, sucrose 70 mmol/L, HEPES 5 mmol/L, and PH 7.4. The mitochondrial isolation procedures were completed within 1 h after the rabbits were euthanized. Mitochondrial protein concentration was quantified using the Bradford colorimetric method.

### Mitochondrial respiration

Mitochondrial respiratory function was measured polarographically at 25 °C using a Clark-type oxygen electrode (Oroboros Instruments, Innsbruck, Austria). In a 2-mL closed thermostatic and magnetically stirred glass chamber, respiration medium containing Mannitol 225 mmol/L, Sucrose 70 mmol/L, EDTANa_2_ 1 mmol/L, KH_2_PO_4_ 20 mmol/L, K_2_HPO_4_ 20 mmol/L, BSA 1 mg/mL (PH, 7.4) was saturated with ambient oxygen to reach a concentration of 258 μmol/L. After an equilibration period, 300 μg mitochondrial protein was added to the reaction system. When the mitochondrial oxygen consumption was stable, 15 μL mixture of 0.8 mol/L malic acid and 1 mol/L glutamic acid was added to initiate the state 2 respiration. After stable state 2 respiration was established, oxidative phosphorylation was started by the addition of 200 nmol/L ADP. Namely, state 3 respiration was initiated. When all of the ADP had been phosphorylated to ATP, state 4 respiration started. The respiratory control ratio (RCR) was calculated as the ratio of the respiratory rate in state 3 to that in state 4.

### Mitochondrial membrane potential

Mitochondrial membrane potential (Δψ) was assessed by tetraethyl benzimidazolyl carbocyanine iodide cationic dye at 37 °C in 2 mL of respiration medium, which exhibited potential-dependent accumulation in mitochondria, resulting in a fluorescence emission shift from 525 nm (green) to 590 nm (red). Theoretically, loss of Δψ was detectable by the decrease in the red to green fluorescence emission ratio. 300 μg mitochondrial protein was added into the respiration medium with tetraethyl benzimidazolyl carbocyanine iodide dye. After equilibration for 10 min, mitochondrial respiratory function was initiated by a 15 μl mixture of 0.8 mol/L malic acid and 1 mol/L glutamic acid, and the alteration of the fluorescence emission was detected.

### Mitochondrial reactive oxygen species production

Mitochondrial ROS generation was assessed using fresh mitochondrial suspensions with the dichlorodihydrofluorescein diacetate probe [19]. Mitochondrial protein 300 μg was added to a quartz cuvette containing 3 mL of phosphate buffer (KCl 130 mmol/L, MgCl_2_ 43 mmol/L, NaH_2_PO_4_ 20 mmol/L, glucose 30 mmol/L, malate 2 mmol/L, PH 7.4) and 2 μL of 2.5 mmol/L dichlorodihydrofluorescein diacetate which was dissolved in 1.25 mmol/L methanol and kept in the dark at 0 °C. The mixture was incubated at 37 °C for 15 min, and dichlorodihydrofluorescein diacetate formation was determined fluorometrically at the excitation wavelength of 499 nm and emission wavelength of 521 nm at 37 °C for 2 min using a Cary Eclipse Fluorescence spectrophotometer (Varian). The dichlorodihydrofluorescein diacetate fluorescence was normalized to the fold of the control group [20].

### Serum biochemical, inflammatory oxidative stress measurements

Blood samples collected were used for serum biochemical examination, including fasting glucose, total cholesterol, triglyceride, low-density lipoprotein cholesterol (LDL-c), high-density lipoprotein cholesterol (HDL-c) and creatinine level by full automatic biochemical analyzer. Fasting insulin and GLP-1 level were assessed using Rabbit Insulin ELISA Kit (Wuhan Huamei Biological Engineering Co., Ltd, Hubei Province, China) and Rabbit Glucagon Like Peptide-1 ELISA Kit (Shanghai Huding Biological Technology Co., Shanghai, China) according to the manufacturer’s instructions respectively. Serum oxidative stress related markers Lipid Peroxidation Malondialdehyde (MDA) and 8-hydroxy-2 deoxyguanosine (8-OHdG) were assessed by MDA Assay Kit (Nanjing Jianchen Bioengineering Institute, Jiangsu Province, China) and Rabbit 8-OHdG ELISA Kit (Shanghai Huding Biological Technology Co., Shanghai, China). Serum antioxidant enzyme superoxide Jianchen Bioengineering Institute, Jiangsu Province, China). And the inflammation maker high-sensitivity C-reactive protein (hs-CRP) level was detected using Rabbit hs-CRP ELISA KIT (Wuhan Huamei Biological Engineering Co., Ltd, Hubei Province, China).

### Histological and ultrastructural analyses

The LV myocardium was cut at 4-µm intervals and stained with Masson’s trichrome stain to evaluate the extent of interstitial fibrosis. Five Masson staining sections from each myocardium were selected, and in each section, five random sampled areas were studied at 400× field of view. Briefly, to quantify the areas of interstitial fibrosis in the LV myocardium, the blue pixel content of the digitized images, excluding the perivascular fibrotic areas, were measured relative to the total tissue area using Image Pro Plus 6.0 Scion image software (Scion Corporation). LV tissues that have been fixed in 2.5% glutaraldehyde for 2 h were used for ultrastructural analysis. After further fixated in 1% osmium tetroxide, dehydrated in ethanol, and embedded in Epon, ultrathin sections were cut from each sample.

Finally, each ultrathin section was counterstained with uranium acetate and lead citrate, and evaluated under H-7650 transmission electron microscope (Hitachi).

### Western blot analysis

Western blotting was performed to assess the expression of transforming growth factor-β1 (TGF-β1) and nuclear factor kappa B (NF-κB) P65, as well as the proteins about mitochondrial biogenesis in three groups. LV tissue protein was extracted by lysis buffer containing 150 mmol/L sodium chloride, 10 mmol/L Tris, 0.01 mol/L EDTA 4 Na, 1% NP40, 10 μg/mL Aprotein, 10 μg/mL leupeptin, 1 mmol/L PMSF, 1 mmol/L Na3VO4, 10 mmol/L NaF. The lysates were centrifuged at 12,000×*g* for 20 min, and the supernatants were collected. The protein content was assayed using a BCA protein assay reagent kit (Thermo Scientific, USA). Total protein was fractionated by electrophoresis and transferred onto PVDF sheets (Millipore, USA) and separately incubated with a specific antibody targeting transforming growth factor-β1 (TGF-β1) (1:5000; Abcam, USA), nuclear factor kappa-light-chain-enhancer of activated B cells (NF-κB) P65 (1:1000; Abcam, USA), Peroxisome proliferator-activated receptor gamma coactivator 1-alpha (PGC-1α) (1:1000; Abcam, USA), transcription of nuclear respiratory factors 1 (NRF1) (1:1000; Abcam, USA), mitochondrial transcription factor A (Tfam) (1:1000; Abcam, USA), followed by incubation with appropriate peroxidase-conjugated secondary antibodies. The reactions were visualized using Tanon 5200 Multi Chemiluminescent Imaging System (Tanon Science & Technology Co., Ltd, Shanghai, China).

### Statistical analysis

Statistical analysis was performed using SPSS 19.0 statistical software. Data were presented as mean ± standard deviation. Comparisons among the three groups were analyzed for statistical significance using the one-way analysis of variance (ANOVA) followed by Bonferroni correction for comparisons between two groups respectively. A *P* value < 0.05 was considered statistically significant.

## Results

### Echocardiographic and hemodynamic studies

**Fig. 1:**
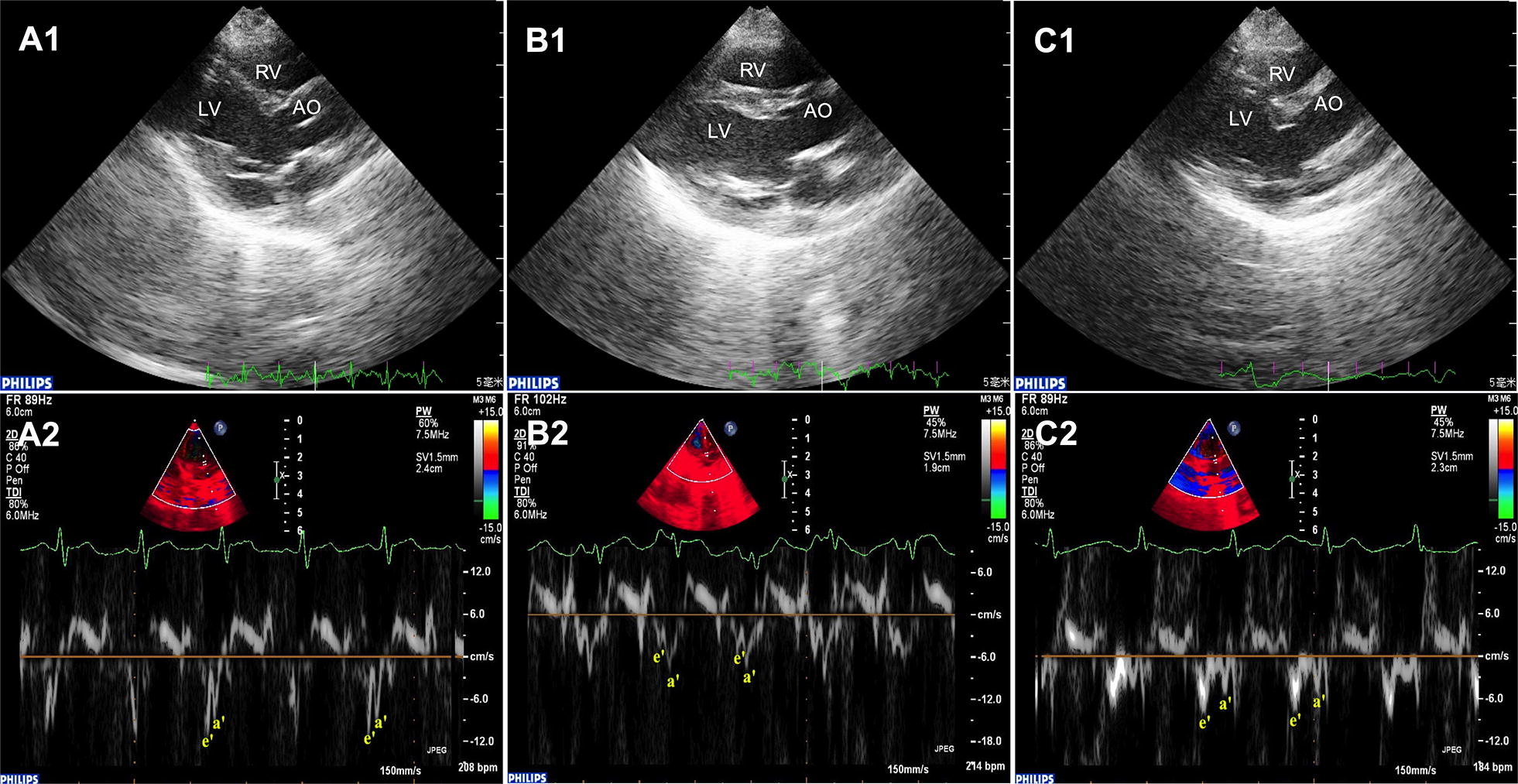
Representative echocardiographic images of the left ventricle for the three different groups. Two-dimensional echocardiographic image of the control group, diabetic group and alogliptin-treated diabetic group (**A**–**C**, top) and Doppler tissue imaging (bottom). *LV* left ventricle, *RV* right ventricle; *AO* aorta, *e′* early diastolic mitral annular velocity, *a′* late diastolic mitral annular velocity, n = 10 per group

Representative echocardiographic images in the parasternal long-axis view and Doppler tissue imaging of the LV from the three groups are shown in Fig. 1A–C with the data shown in Table 1. Compared with the control group, the left atrial diameter, interventricular septum diameter and left ventricular posterior wall diameter were significantly increased in the diabetes group (*P* < 0.05 or 0.01). These changes were partially prevented by alogliptin (*P* < 0.05 or 0.01). By contrast, left ventricular ejection fraction was not significantly different between the three groups. Nevertheless, E/e′ ratio, a marker of left ventricular diastolic function, was increased in the diabetic group (*P* < 0.01) and reduced by alogliptin (*P* < 0.05).

**Table 1:** Hemodynamic and echocardiographic studies

	CON group (n = 10)	DM group (n = 10)	DM-A group (n = 10)	*P* value
HR (bpm)	281.4 ± 10.2	291.5 ± 11.3	279.8 ± 15.0	0.145
SBP (mmHg)	124.38 ± 5.95	128.99 ± 5.83	128.80 ± 5.49	0.217
DBP (mmHg)	78.03 ± 4.19	81.05 ± 4.72	80.6 ± 5.71	0.428
MBP (mmHg)	95.4 ± 4.53	97.79 ± 9.93	98.55 ± 5.70	0.658
LVEDP (mmHg)	0.18 ± 0.07	2.37 ± 0.57**	1.07 ± 0.30**^##^	< 0.001
+ dp/dtmax (mmHg/m)	3969.02 ± 464.27	3643.68 ± 332.94	3994.28 ± 380.08	0.167
− dp/dtmax (mmHg/m)	2852.58 ± 435.33	1475.75 ± 266.74**	2154.03 ± 391.43**^##^	< 0.001
LAD (mm)	6.25 ± 0.90	8.94 ± 0.88**	7.81 ± 0.60**^#^	< 0.001
IVS (mm)	1.83 ± 0.31	2.75 ± 0.33**	2.27 ± 0.17*^##^	< 0.001
PWLV (mm)	1.71 ± 0.37	2.74 ± 0.32**	2.26 ± 0.19**^#^	< 0.001
LVEDD (mm)	11.92 ± 0.72	12.82 ± 0.97	12.19 ± 0.71	0.097
LVESD (mm)	7.79 ± 0.53	7.58 ± 0.57	7.97 ± 0.64	0.426
LVEF (%)	56.31 ± 2.50	55.94 ± 2.59	56.40 ± 1.76	0.916
RVEDD (mm)	4.61 ± 0.71	4.98 ± 0.55	4.99 ± 0.38	0.325
E/e′ ratio	4.95 ± 1.36	11.73 ± 2.85**	8.89 ± 1.16**^#^	< 0.001
Heart weight ratio (1/1000)	2.58 ± 0.19	2.82 ± 0.11	2.68 ± 0.24	0.054
Body weight (kg)	3.42 ± 0.30	2.72 ± 0.75**	2.95 ± 0.26**	< 0.001

Values are mean ± SD

HR, heart rate; SBP, systolic blood pressure; DBP, diastolic blood pressure; MBP, mean blood pressure; LVEDP, left ventricular end diastolic presssure; + dp/dtmax, maximal increasing rate of left intraventricular pressure; − dp/dtmax, maximal decreasing rate of left intraventricular pressure; LAD, left atrial diameter; IVS, interventricular septal; PWLV, left ventricular posterior wall; LVEDD, left ventricular end-diastolic dimension; LVESD, left ventricular end-systolic dimension; LVEF, left ventricular ejection fraction; RVEDD, right ventricular end-diastolic dimension; E/e′: the ratio of early diastolic filling velocity to the early diastolic mitral annulus velocity

* Compared with CON group, P < 0.05

** Compared with CON group, P < 0.01

^#^ Compared with DM group, P < 0.05

^##^ Compared with DM group, P < 0.01

Hemodynamic studies revealed that left ventricular end-diastolic pressure were markedly increased in the diabetes group (*P* < 0.01), changes that were partially prevented by alogliptin treatment (*P* < 0.01). No difference in the maximal pressure differential increase (+ dp/dtmax) among the three groups. However, the maximal pressure differential decrease (− dp/dtmax) was lower in the diabetes group and reversed by alogliptin (*P* < 0.01).

### Mitochondrial respiratory function, membrane potential, and ROS generation

**Fig. 2:**
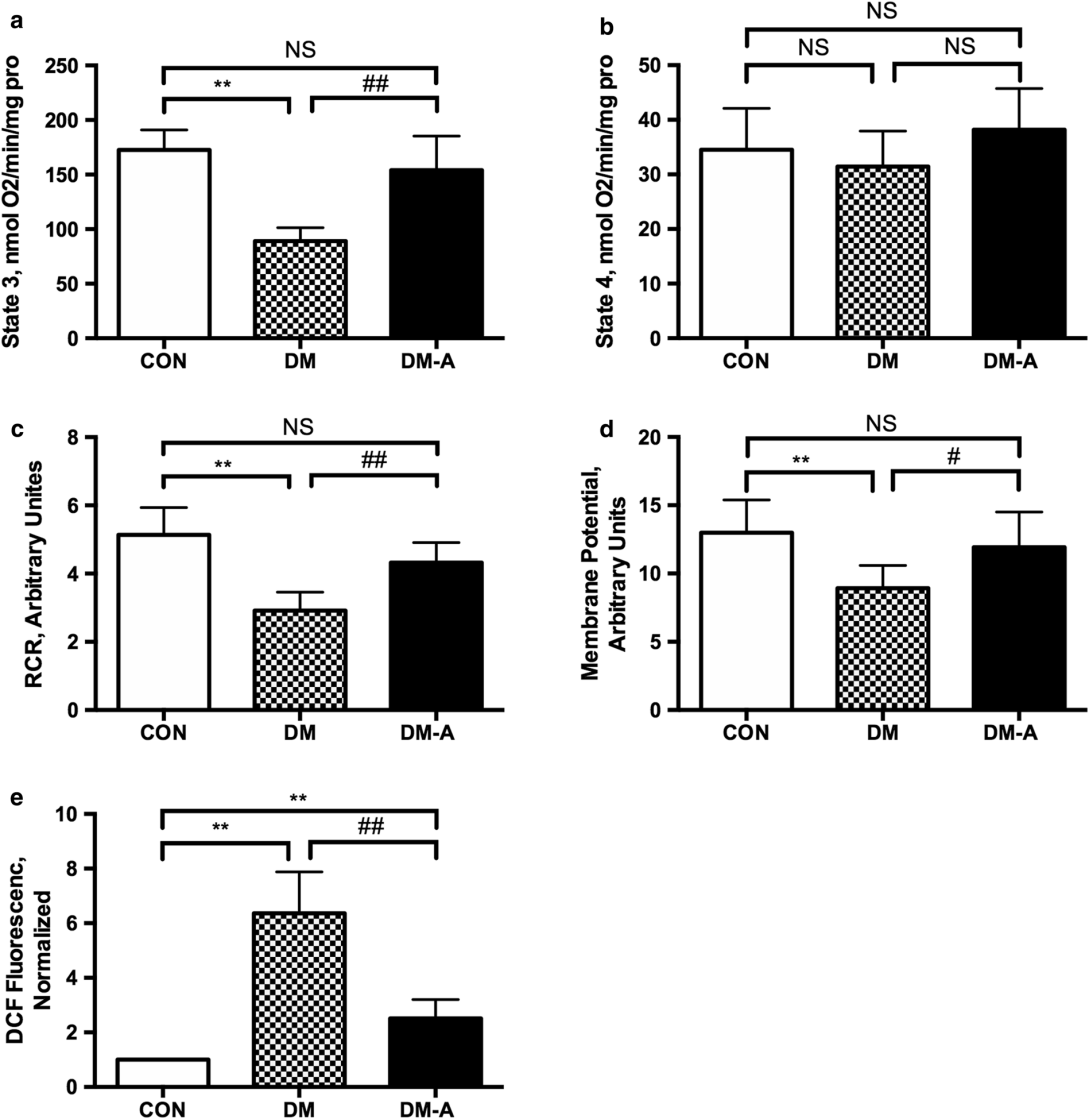
Effects of alogliptin on mitochondrial state 3 respiration rate (**a**), state 4 respiration rate (**b**), respiratory control ratio (RCR) (**c**), mitochondrial membrane potential (**d**) and ROS levels (**e**). *CON* control group, *DM*: diabetic group, *DM-A* alogliptin-treated diabetic group. **P < 0.01 versus CON group; ^#^*P* < 0.05 versus DM group; ^##^*P *< 0.01 versus DM group. n = 10 per group

Subsequent experiments examined mitochondrial respiratory function, membrane potential and generation of reactive oxygen species. Thus, state 3 respiration rate was lower in the diabetes group, which was restored by alogliptin treatment (*P *< 0.01; Fig. 2a). No significant difference of state 4 respiration rate was observed among the three groups (Fig. 2b). Consequently, the respiratory control ratio, defined as the rate ratio of state 3 to state 4 respiration, was higher in the diabetes group (*P *< 0.01; Fig. 2c) and subsequently lower by alogliptin. The diabetes group showed lower mitochondrial Δψ (*P *< 0.05; Fig. 2d) and higher mitochondrial ROS generation rate compared to the control group, changes that were reversed by alogliptin treatment (*P *< 0.01; Fig. 2e).

### Serum biochemical and oxidative stress-related markers

Fasting glucose at week 12 was higher (*P *< 0.01) and levels of insulin and GLP-1 were lower (*P *< 0.01) in the diabetic group compared with controls (Table 2). Serum creatinine, triglycerides, total cholesterol, and low- and high-density lipoprotein cholesterol, were not significantly different between the three groups. By contrast, higher MDA, 8-OHdG, and high-sensitivity C-reactive protein concentrations (*P *< 0.01) and lower superoxide dismutase activity (*P *< 0.01) were observed in the diabetic group (*P *< 0.01). Alogliptin treatment for 12 weeks led to partial restoration of fasting glucose and GLP-1 levels (*P *< 0.01: compared to DM group), and attenuated these inflammatory and oxidative stress markers (*P *< 0.01) with the exception for superoxide dismutase activity.

**Table 2:** Serum biochemical, oxidative stress and inflammation examination

	CON group (n = 10)	DM group (n = 10)	DM-A group (n = 10)	*P* value
Glucose (mmol/L)	4.98 ± 0.45	14.12 ± 1.69**	11.03 ± 2.01**^#^	< 0.001
INS (mmol/L)	17.84 ± 2.57	9.06 ± 1.43**	11.45 ± 1.16**^#^	< 0.001
GLP-1 (pmol/L)	1.28 ± 0.35	0.42 ± 0.16**	0.90 ± 0.15*^##^	< 0.001
Cr (μmol/L)	80.90 ± 15.28	92.36 ± 15.07	89.31 ± 8.53	0.229
TC (mmol/L)	1.43 ± 0.29	1.66 ± 0.34	1.73 ± 0.29	0.143
TG (mmol/L)	1.15 ± 0.23	1.21 ± 0.37	1.16 ± 0.27	0.997
LDLc (mmol/L)	0.30 ± 0.14	0.37 ± 0.12	0.35 ± 0.13	0.576
HDLc (mmol/L)	0.44 ± 0.11	0.47 ± 0.07	0.45 ± 0.11	0.827
SOD (U/mL)	476.52 ± 61.76	386.28 ± 52.86**	408.68 ± 28.17*	0.004
hs-CRP (mg/L)	1.49 ± 0.68	6.19 ± 1.63**	3.27 ± 1.04*^##^	< 0.001
MDA (nmol/mL)	9.51 ± 2.00	16.62 ± 2.77**	12.49 ± 2.13^##^	< 0.001
8-OHdG (ng/mL)	1.51 ± 0.50	3.79 ± 0.70**	2.27 ± 0.26*^##^	< 0.001

Values are mean ± SD

INS, insulin; GLP-1, glucagon like peptide-1; Cr, creatinine; TC, total cholesterol; TG, triglyceride; LDLc, low-density lipoprotein cholesterol; HDLc, high-density lipoprotein cholesterol; SOD, superoxide dismutase; hs-CRP, hypertensive C-reactive protein; MDA, malondialdehyde; 8-OHdG, 8-hydroxy-2′-deoxyguanosine

* Compared with CON group, P < 0.05

** Compared with CON group, P < 0.01

^#^ Compared with DM group, P < 0.05

^##^ Compared with DM group, P < 0.01

### Left ventricular interstitial fibrosis and cardiomyocyte ultrastructure

**Fig. 3:**
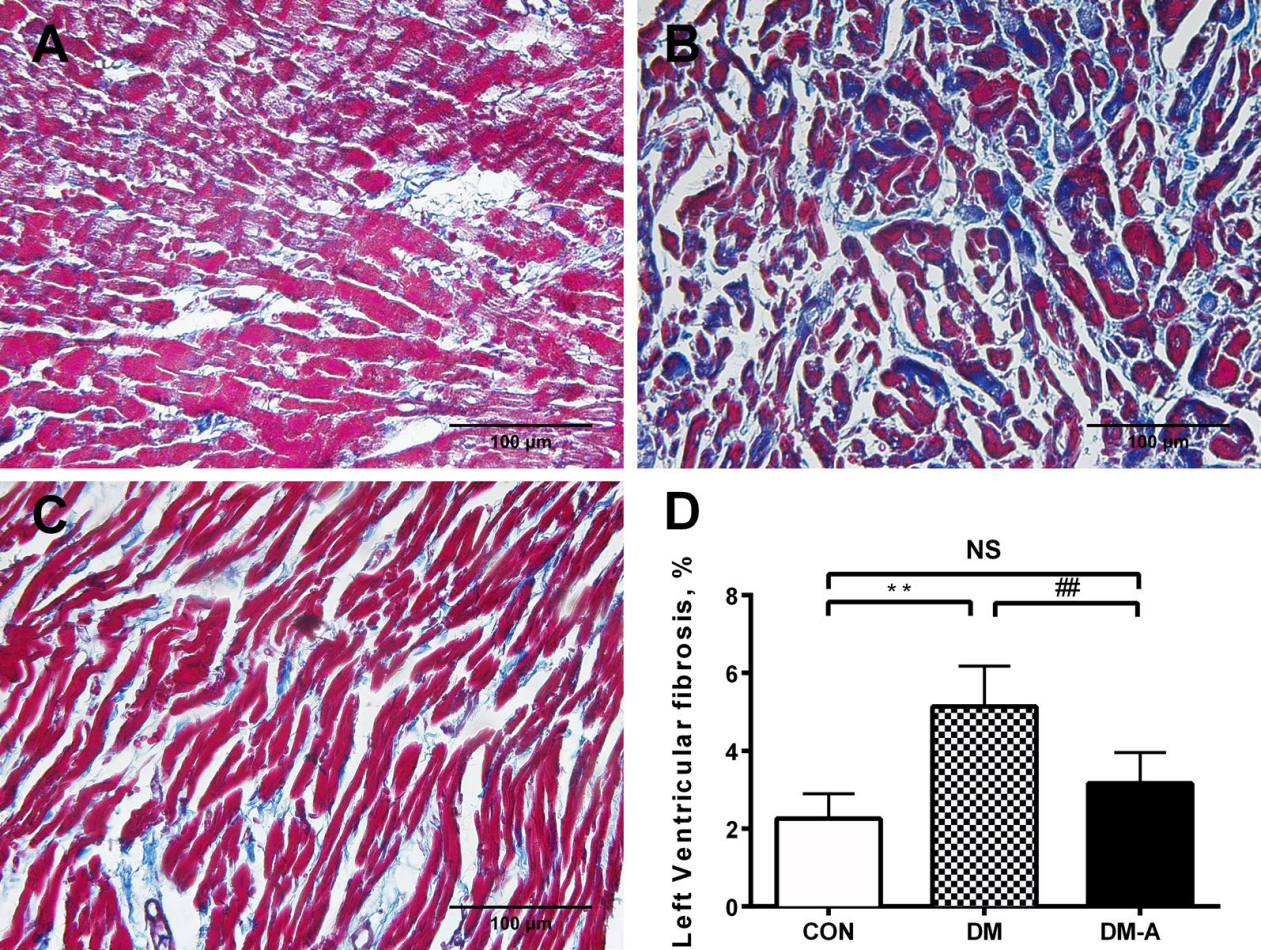
Left ventricular interstitial fibrosis in the control group, diabetic group and alogliptin-treated diabetic group (**A**–**C**). Area of fibrosis to the area of the reference tissue (**D**). **Compared with CON group, *P *< 0.01; ^##^Compared with DM group, *P *< 0.01. n = 6 per group

**Fig. 4:**
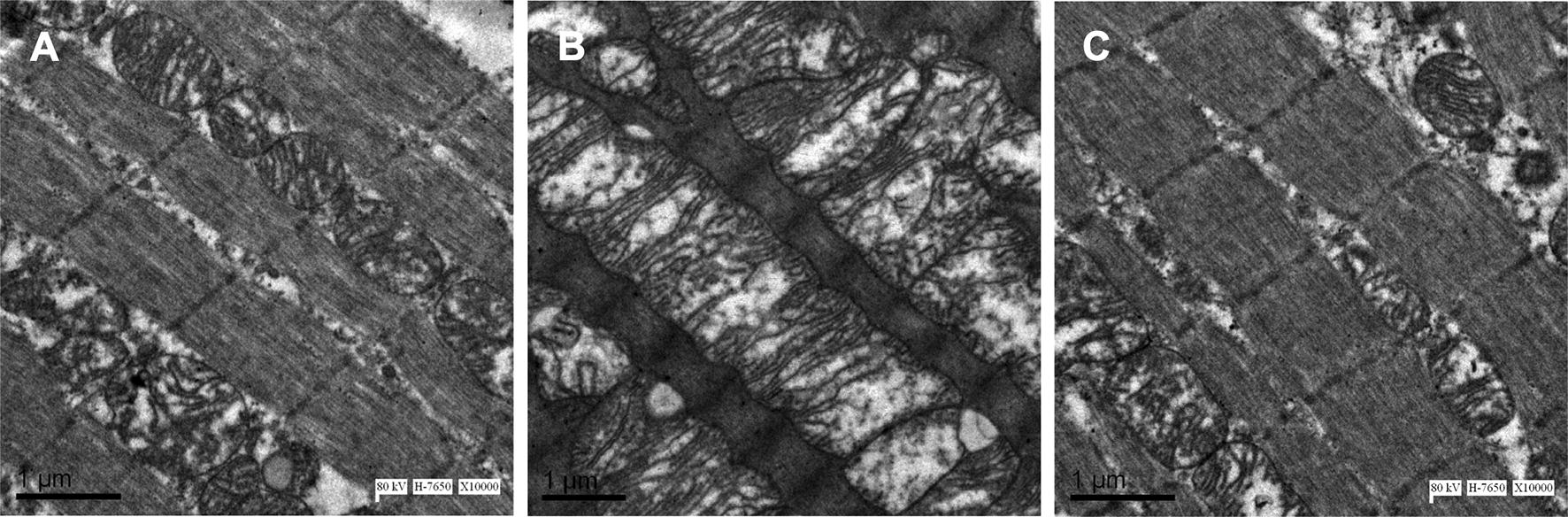
Comparison of left ventricular ultrastructure in the control group, diabetic group and alogliptin-treated diabetic group (**A**–**C**). n = 4 per group

Representative histological sections from the left ventricle are shown in Fig. 3A–C. Extensive interstitial fibrosis was observed in the diabetic group compared to controls, which was attenuated by alogliptin treatment. The ultrastructure of left ventricular cardiomyocytes under the different conditions are shown in Fig. 4A–C. There were regular sarcomere organization and uniformly-sized mitochondria in the control group. Under the diabetic condition, swelling mitochondria between sarcomeres were observed. These changes were prevented by alogliptin treatment.

### Protein expression levels of transforming growth factor-β1, nuclear factor κB p65 and mitochondrial biogenesis-related proteins

**Fig. 5:**
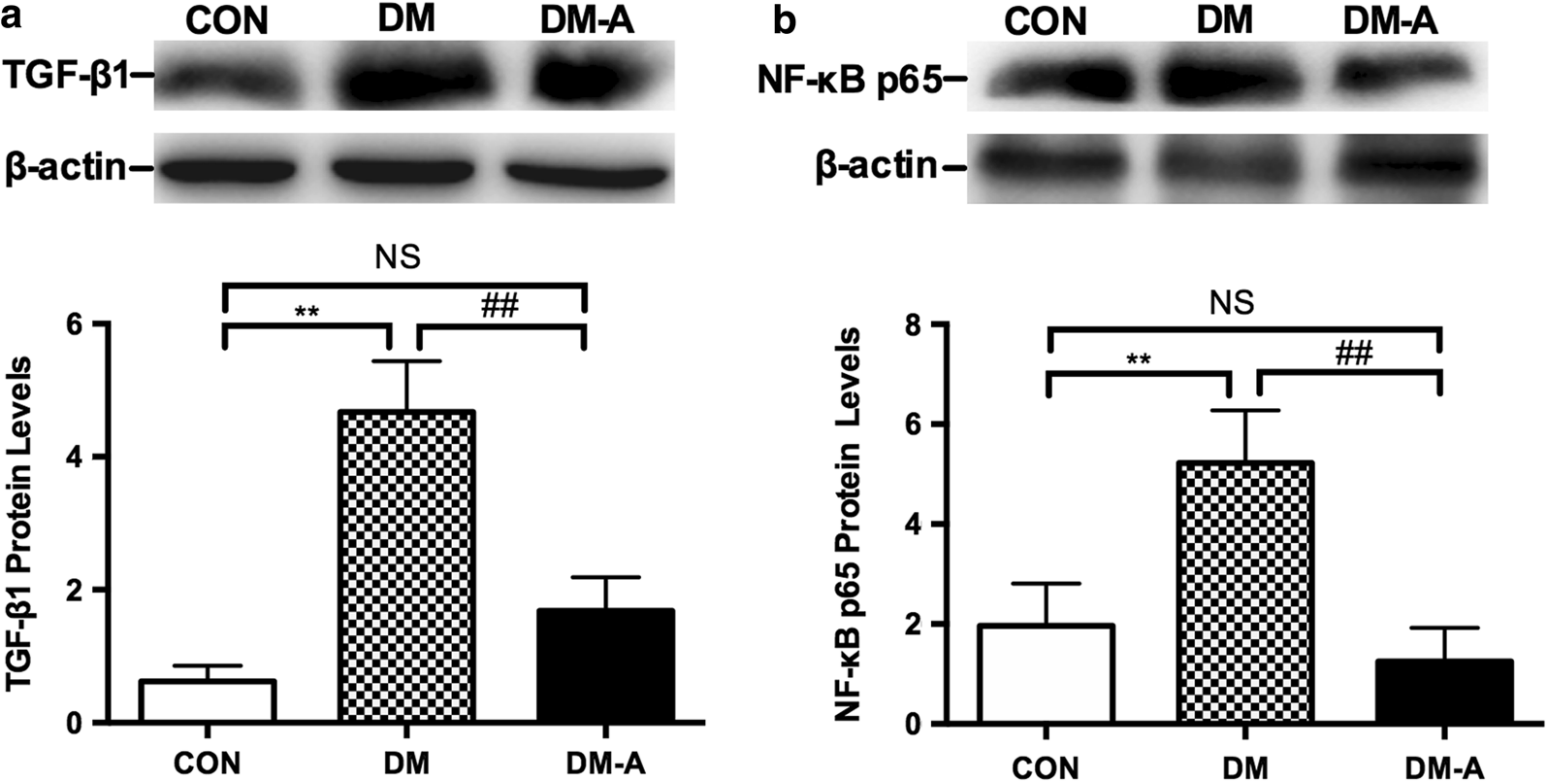
TGFβ1 (**a**) and NF-κB p65 protein (**b**) expression in left ventricular tissue by Western blot analysis. **P < 0.01 versus CON group; ^##^*P *< 0.01 versus DM group. n = 3 to 6 per group

**Fig. 6:**
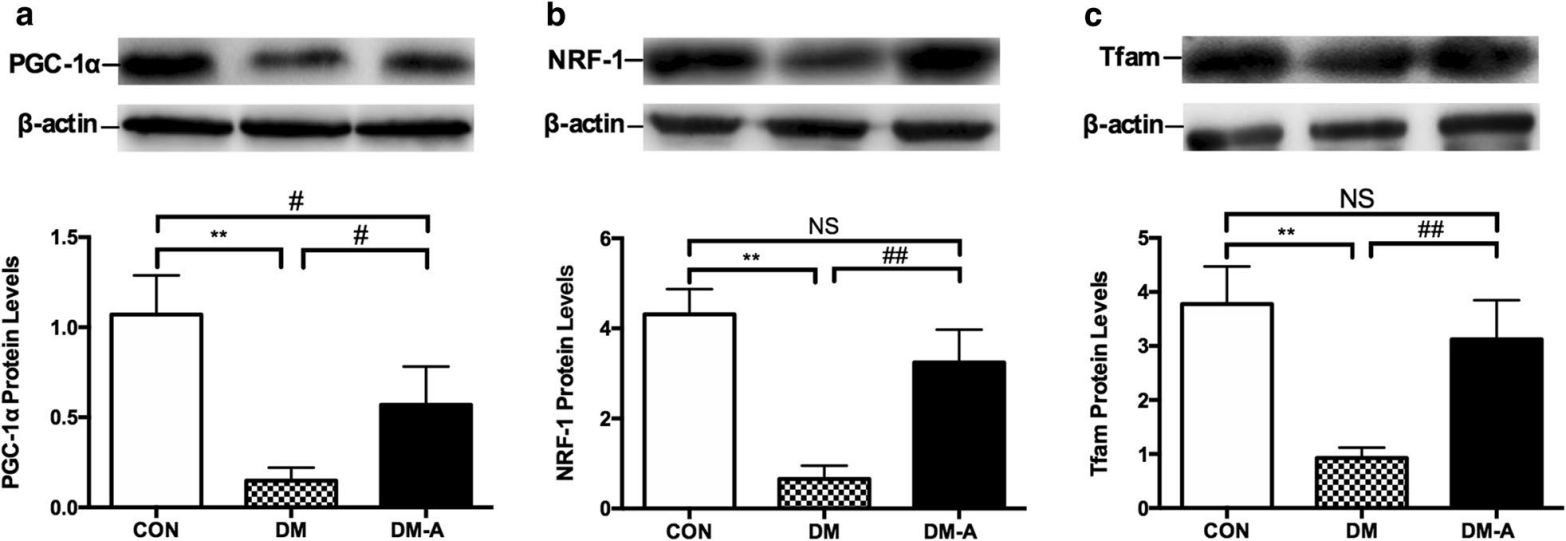
Protein expression levels of mitochondrial biogenesis-related proteins in left ventricular tissue estimated by Western blot analysis. **a**–**c** PGC-1α, NRF-1, and Tfam protein levels. **P < 0.01 versus CON group; ^#^*P* < 0.05 versus DM group; ^##^*P *< 0.01 versus DM group. n = 3 to 6 per group

Protein expression levels of transforming growth factor β1 (TGF-β1) (Fig. 5a) and nuclear factor kappa B (NF-κB) p65 (Fig. 5b) were significantly higher in the diabetic group compared to controls (*P *< 0.01). These changes were fully reversed by alogliptin treatment (*P *< 0.01). By contrast, protein expression levels of the mitochondrial biogenesis-related proteins, PGC-1α (Fig. 6a), NRF-1 (Fig. 6b), and Tfam (Fig. 6c) were significantly lower in the diabetic group (*P *< 0.01). Alogliptin treatment partially restored the expression of these proteins.

## Discussion

In this study, we demonstrated that the DPP-4 inhibitor alogliptin prevented diastolic dysfunction and attenuated left ventricular remodeling in an alloxan-induced diabetic rabbit model. The advantages of the rabbit model include a longer lifespan compared to smaller animals, relatively ease of handling and low costs [21]. In our previous work, we found that left atrium showed significant structural remodeling and a trend towards lower LV − dp/dtmax, which reflects ventricular wall stiffness in the diabetes group at 8 weeks [14]. In this study, we studied cardiac function over a longer period of 12 weeks, demonstrating the presence of diastolic dysfunction (higher E/e′) with normal systolic function in the diabetic state, associated with increased left ventricular end-diastolic pressure and decreased − dp/dtmax.

### Effects of different DPP-4 inhibitors on diastolic function

DPP-4 inhibitors have been increasingly used for hyperglycemic control in type 2 diabetes with good tolerance [22] although its benefit in type 1 diabetes is controversial [23]. It acts by enhancing the level of GLP-1 and activating its receptor. Independently of glycemic control, DPP-4 inhibitors exert cardiovascular protection effect probably through their anti-inflammatory [24], anti-fibrosis [25] and anti-oxidant activities [26]. It has been shown that DPP-4 inhibitors decrease plasma and brain oxidative stress levels in high-fat diet-induced insulin-resistant rats, and restored impaired brain mitochondrial function [27].

Some clinical studies have found improvements in cardiovascular outcomes in diabetes with the use of DPP-4 inhibitors. For example, Yamada et al. [28] found that the addition of sitagliptin to conventional diabetic care significantly improve diastolic function. Data from pre-clinical experiments have provided evidence for their mechanisms underlying cardiac protective effects [29, 30], including reduced lipolysis [31], inhibition of TRAF3IP2 expression and its downstream inflammatory signaling [32], reduced activation of mTOR/S6K1 and reduced IRS-1 and IRS-2 degradation [33].

### Oxidative stress and diastolic dysfunction in the diabetic heart

The mechanisms underlying diastolic dysfunction in diabetes include left ventricular interstitial fibrosis leading to an increase in passive stiffness and abnormal myocardial active relaxation due to structural remodeling and metabolic derangements [34]. Hyperglycemia promotes cardiomyocyte dysfunction because of metabolic alterations involving glucose and lipid handling [35, 36]. Furthermore, it promotes the generation of advanced glycation end-products (AGEs), inflammation, and oxidative stress may directly activate resident cardiac fibroblasts and induce a matrix-synthetic phenotype by the activation of fibrogenic growth factors, especially TGF-β1 as shown in this study [37]. Overproduction of ROS leads to increased formation of AGEs, activation of the AGE receptor (RAGE) and protein kinase C (PKC) in the polyol and hexosamine pathways [12, 38, 39], in turn perpetuating electrophysiological and structural remodeling [40–42]. In our study, the content of lipid oxidation products MDA and DNA oxidation products 8-OHdG were markedly raised in diabetic rabbits. Consistent with previous reports [43, 44], we found that DPP-4 inhibitors have favorable effects in inhibiting oxidative stress, ameliorating ventricular remodeling and improving diastolic dysfunction.

### Mitochondrial abnormalities contribute to diastolic dysfunction in diabetes

NADPH oxidases, lipoxygenases, and xanthine oxidase are major systems that generate endogenous ROS [45, 46]. Mitochondria are thought to be the major contributor of this oxidative stress during oxidative phosphorylation [47]. Mitochondrial oxidative stress itself can promote further mitochondrial dysfunction through ROS-dependent ROS release [48]. This can result in Δψ collapse, cytochrome c release, mitochondrial swelling, and cellular apoptosis [49]. Finally, mitochondrial biogenesis is a dynamic and tightly regulated process that is essential for maintenance of its quantity and function. Recent studies have reported that mitochondrial biogenesis was impaired in cardiomyocytes in diabetes [50]. PGC-1α is a critical regulator of mitochondrial biogenesis through its downstream mediators NRF-1 and Tfam [51]. It also regulates calcium signaling and calcium mediated cell death [52].

The relationship between mitochondrial dysfunction and diastolic dysfunction has been explored in non-diabetes models [53]. The novelty of this study is the demonstrations that DPP-4 inhibitors decreased mitochondrial ROS production rate, prevented mitochondrial membrane depolarization, alleviated mitochondrial swelling, and increased mitochondrial respiration function. In addition, alogliptin restored the protein expression levels of PGC-1α, NRF-1 and Tfam that were reduced by diabetes. It was reported that silent information regulator 1 (sirt1) activation by the agent resveratrol protected against diabetes-related cardiac dysfunction by improving mitochondrial biogenesis via the PGC-1α pathway [54].

## Study limitations

Several limitations of the present study should be acknowledged. Firstly, as we only used one medication of the DPP-4 inhibitor class, further studies are needed whether the effects of alogliptin represent a class effect. Secondly, we did not examine calcium handling and cellular apoptosis, which could contribute to the pathogenesis of diabetic cardiomyopathy. Thirdly, the extent to which the angiotensin II pathway, which is known to induce fibrosis, played a role remains uncertain. Fourthly, C_max_ of alogliptin was not measured in our study. Moreover, biomarkers such as B-type natriuretic protein and advanced glycation products, and parameters such as Hba1c, were also not measured. Finally, it is unclear the extent to the improved diastolic function can be attributed to better glycemic control. Further studies can examine whether electrophysiological abnormalities observed in diabetes can be prevented by alogliptin.

## Conclusions

The DPP-4 inhibitor alogliptin prevents cardiac diastolic dysfunction by inhibiting ventricular remodeling, explicable by improved mitochondrial function and increased mitochondrial biogenesis.

## Abbreviations

DBP: diastolic pressure
DM: diabetes mellitus
DPP-4: dipeptidyl peptidase-4
HDL-c: high-density lipoprotein cholesterol
HR: heart rate
hs-CRP: high-sensitivity C-reactive protein
IVST: interventricular septal thickness
LDL-c: low-density lipoprotein cholesterol
LVEDD: left ventricular end-diastolic dimension
LVESD: left ventricular end-systolic dimension
LVEF: left ventricular ejection fraction
LVEDP: left ventricle end-diastolic pressure
LVPWT: left ventricular posterior wall thickness
MBP: mean pressure
MDA: malondialdehyde
mTOR: mechanistic target of rapamycin complex 1
NRF1: transcription of nuclear respiratory factors 1
PGC-1α: PPAR co-activator α
RCR: respiratory control ratio
ROS: reactive oxygen species
SBP: systolic pressure
SOD: superoxide dismutase
S6K1: ribosomal protein S6 kinase beta-1
Tfam: mitochondrial transcription factor A
TRAF3IP2: TRAF3 Interacting Protein 2
Δψ: mitochondrial membrane potential
8-OHDG: 8-hydroxy-2 deoxyguanosine
